# Moment-Based Parameter Estimation for the Γ-Parameterized TWDP Model

**DOI:** 10.3390/s22030774

**Published:** 2022-01-20

**Authors:** Pamela Njemcevic, Enio Kaljic, Almir Maric

**Affiliations:** Department of Telecommunications, Faculty of Electrical Engineering, University of Sarajevo, Zmaja od Bosne b.b., Kampus Univerziteta, 71000 Sarajevo, Bosnia and Herzegovina; enio.kaljic@etf.unsa.ba (E.K.); almir.maric@etf.unsa.ba (A.M.)

**Keywords:** TWDP fading channel, moment-based estimation, Cramer–Rao bound, asymptotic variance

## Abstract

Two-wave with diffuse power (TWDP) is one of the most promising models for description of a small-scale fading effects in the emerging wireless networks. However, its conventional parameterization based on parameters K and Δ is not in line with model’s underlying physical mechanisms. Accordingly, in this paper, we first identified anomalies related to usage of conventional TWDP parameterization in moment-based estimation, showing that the existing Δ-based estimators are unable to provide meaningful estimates in some channel conditions. Then, we derived moment-based estimators of recently introduced physically justified TWDP parameters K and Γ and analyzed their performance through asymptotic variance (AsV) and Cramer–Rao bound (CRB) metrics. Performed analysis has shown that Γ-based estimators managed to overcome all anomalies observed for Δ-based estimators, simultaneously improving the overall moment-based estimation accuracy.

## 1. Introduction

Small-scale fading severely degrades performance of wireless communication systems [1]. Thus, in order to design highly reliable and efficient transceivers for communication in deep fading conditions, it is of profound significance to have an accurate and tractable fading model [2,3]. Traditionally, the signal affected by small-scale fading in nonline-of-sight (NLOS) environments has been modeled as a sum of many diffuse components and described by Rayleigh distribution. Withal, the Rician fading model has been used for the description of received signal variations in better-than-Rayleigh channel conditions, since, except for many diffuse components, it also assumes the presence of one dominant specular component [4]. However, in some multipath sparse channels, traditional fading models fall short of accurately describing small-scale variations of the received complex envelope [4], which makes them nonviable for accurate modeling of all propagation conditions [5].

The aforementioned is especially pronounced in mmWave 5G communication networks equipped with directional antennas or arrays [6] as well as in wireless sensor networks deployed in cavity environments [3], where measurements have shown that the signal in multipath sparse propagation conditions may, in some cases, experience fading with characteristics worse than those in Rayleigh channels [4].

In such scenarios, the TWDP fading model—which assumes the presence of two specular components instead of just one considered by Rician model—appears to be appropriate for modeling small-scale fading effects [7]. In this regard, it is shown that TWDP distribution describes small-scale fading more accurately than conventional distributions in static sensor networks with their nodes placed within cavity environments, such as aircrafts and buses [3] and abroad a large transport helicopter [8]. It is also shown that its PDF shape significantly coincides with empirical results obtained for train-to-infrastructure wireless communication [9], vehicle-to-vehicle 60 GHz urban communication [10], and mmWave communication in indoor environments [11,12].

Thereat, the TWDP model itself represents generalization of Rayleigh and Rician fading models and can be used for modeling both better-than-Rayleigh and worse-than-Rayleigh fading conditions. It is based on the assumption that the waves arriving at the receiver can be observed as a sum of two specular components with constant magnitudes $V_{1}$ and $V_{2}$ and uniformly distributed phases, plus many diffuse components treated as a complex zero-mean Gaussian random process with average power $2\sigma^{2}$. As such, the model is conventionally parameterized by *K* and Δ, defined as [13]:

(1) $$\begin{array}{c} K=\frac{V_{1}^{2}+V_{2}^{2}}{2\sigma^{2}} \end{array}$$

(2) $$\begin{array}{c} \Delta =\frac{2V_{1}V_{2}}{V_{1}^{2}+V_{2}^{2}} \end{array}$$

where parameter *K* (K≥0) characterizes the ratio of the average power of specular components to the average power of remaining diffuse components (like the Rician parameter $K_{Rice}=V_{1}^{2}/\left(2\sigma^{2}\right)$) and parameter Δ(0≤Δ≤1) characterizes the relation between magnitudes of specular components. Consequently, the average received signal power Ω is equal to $V_{1}^{2}+V_{2}^{2}+2\sigma^{2}$.

However, as it is elaborated in [14], the definition of parameter Δ is not in accordance with the model’s underlying physical mechanisms. Namely, according to the model’s definition, specular components have constant magnitudes and are propagating in a linear medium. Consequently, $V_{2}$ can be nothing but the linear combination of $V_{1}$, wherefore the function which characterizes the ratio between $V_{1}$ and $V_{2}$ has to be linear [14]. However, Δ-based parameterization introduces a nonlinear relation between magnitudes of specular components, since $V_{2}=V_{1}\left(1-\sqrt{\left(1-\Delta^{2}\right)}\right)/\Delta$. This hinders accurate observation of the impact of the ratio between $V_{1}$ and $V_{2}$ on a system’s performance metrics and causes anomalies within the expressions obtained by integration or derivation with respect to parameter Δ [14].

Considering that previously mentioned, Γ-based parameterization was recently proposed in [14], by introducing parameters *K* and Γ:

(3) $$\begin{array}{c} K=\frac{V_{1}^{2}+V_{2}^{2}}{2\sigma^{2}} \end{array}$$

(4) $$\begin{array}{c} \Gamma =\frac{V_{2}}{V_{1}} \end{array}$$

where Γ (0≤Γ≤1, for $0\leq V_{2}\leq V_{1})$ obviously ensures linear dependence between $V_{1}$ and $V_{2}$. On the other side, the definition of parameter *K* remains unchanged, but the definition expression of normalized $K/K_{Rice}$ with respect to the ratio between $V_{1}$ and $V_{2}$, e.g., $K/K_{Rice}=1+{\left(V_{2}/V_{1}\right)}^{2}$ (where $K_{Rice}$ is the Rician parameter), is affected by the choice of second parameter (Δ vs. Γ). Obviously, parameter Δ completely changes its character since $K/K_{Rice}=2\left(1-1\sqrt{1-\Delta^{2}}\right)/\Delta^{2}$, while Γ does not, since $K/K_{Rice}=1+\Gamma^{2}$ [14].

Despite that aforementioned, Γ-based parameterization has up to now been considered only in [14], by elaborating its benefits on ASEP observation accuracy. However, no other benefits of Γ-based parameterization or the anomalies caused by the nonphysical definition of a Δ parameter within the expressions which involve integration or derivation with respect to Δ have been presented yet. Accordingly, in this paper, one such anomaly is identified within Δ-parameterized TWDP moment-based estimators and overcome by introducing the appropriate estimators for Γ-parameterized TWDP fading model. In that sense, this paper focuses on performance analysis of moment-based parameter estimators, for both Δ- and Γ-based TWDP parameterizations.

Considering that aforementioned, in Section 2, an overview of the results related to estimation of TWDP parameters is foremost presented, elaborating in detail anomalies caused by conventional Δ-based parameterization and indicating the absence of those related to a Γ-parameterized TWDP model. In Section 3, a closed-form moment-based estimator is derived for Γ-based parameterization, and their behavior is examined for various values of proposed parameters. In Section 4, performance analysis of proposed estimators is performed. First, corresponding asymptotic variances are derived and graphically presented and then the limits of estimation problem are explored and determined in terms of Cramer–Rao bound. Finally, Δ- and Γ-based estimators are compared in Section 5, in order to gain insight into the differences in their behavior and their estimation accuracy. Conclusions are outlined in Section 6.

## 2. Related Works

The estimation of parameters that characterize fading channel is of practical importance in a variety of wireless scenarios. It includes not only delay insensitive channel characterization and link budget calculations, but also online adaptive coding/modulation for which the estimation of parameters must be both accurate and prompt [15]. In that sense, inaccurate estimation of channel parameters leads to a non-optimal channel capacity utilization, which might significantly affect highly mobile communication systems such as vehicular-to-vehicular and vehicular-to-infrastructure. Accordingly, different approaches in estimation of TWDP parameters are proposed to make a deal between their computational complexity and estimation accuracy. These approaches usually assume that the value of parameter Ω can be directly estimated from the data set as a second moment [10], so the estimation problem has become focused on determination of values of *K* and Δ.

Among the investigated approaches used to estimate parameters of Δ-based parameterization, the distribution fitting approach is used for measurements performed in air-craft and buses at 2.4 GHz [3], while the maximum likelihood procedure (ML) is used for measurements performed at 60 GHz in the indoor environment [11] and in vehicular-to-vehicular propagation scenarios [10]. However, it is shown that both approaches are computationally very complex and inappropriate for online applications. Accordingly, the moment-based approach is considered in [4,7,12], as a compromise between estimator’s complexity and its accuracy. Thereat, in [7], estimators are derived only as conditional expressions which can not be used for practical estimations in which both parameters are unknown. To overcome the issue, the exact joint estimators of TWDP parameters are derived in [4] as computationally simple expressions. However, for certain combinations of parameters *K* and Δ, the estimator of parameter Δ, $\hat{\Delta }$, derived in [4], provides physically unsubstantiated results, which can be demonstrated by applying ([4], Equation (16)) on Monte Carlo simulated samples.

**Figure 1:**
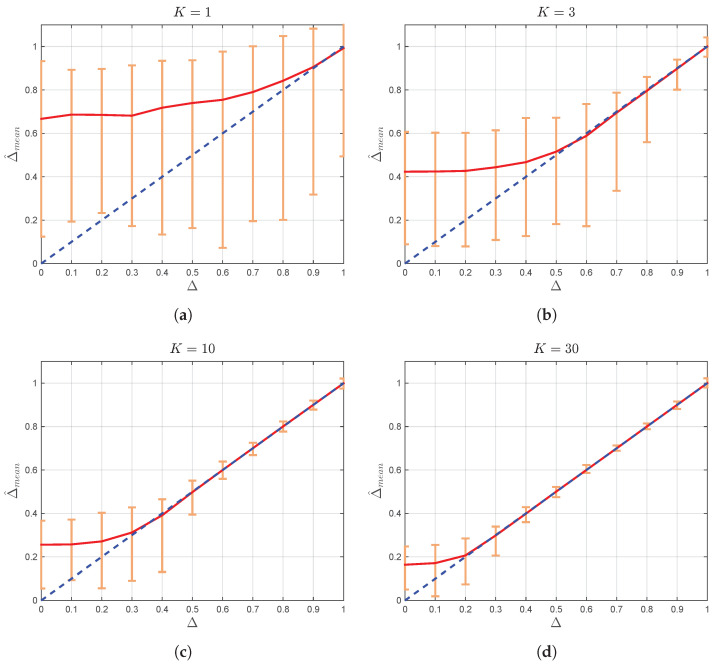
${\hat{\Delta }}_{mean}$ (with absolute error bars) vs. Δ for (**a**) K=1, (**b**) K=3, (**c**) K=10, and (**d**) K=30. The solid line shows a linear regression fit to the data. The unit slope dashed line is illustrated as a benchmark.

Accordingly, for each combination of *K* and Δ, where K={1,3,10,30} and Δ={0,0.1,0.2,…,1}, 500 realizations of the TWDP process with $N=10^{4}$ i.i.d. samples are generated. The estimate of parameter Δ from *j*-th realization, ${\hat{\Delta }}_{j}$, is obtained by ([4], Equation (16)) and, for each combination of *K* and Δ, sample mean value ${\hat{\Delta }}_{mean}$ is calculated as $\left(1/500\right)\sum_{j=1}^{500}{\hat{\Delta }}_{j}$. The results are presented in Figure 1.

From Figure 1, it can be observed that, for large values of Δ (i.e., for Δ≈ 1), Δ estimates may exceed 1 regardless of the value of parameter *K*. However, according to definition of Δ:

(5) $$\begin{array}{cc} \left(1-\Delta \right) & =\frac{{\left(V_{1}-V_{2}\right)}^{2}}{V_{1}^{2}+V_{2}^{2}}\;\;\;\;\;\;\;\;\;\;\;\;\;\;\; \end{array}$$

(6) $$\begin{array}{cc} \left(1-\Delta \right) & \geq 0,\;\mathrm{for}\;\left(V_{1},V_{2}\right)\in R^{2} \end{array}$$

the parameter has to be lower or equal to one (Δ≤1) for any $\left(V_{1},V_{2}\right)\in R^{2}$. Accordingly, estimates of Δ greater than 1 are physically unsubstantiated and can not be used to gain any insight into the relation between $V_{1}$ and $V_{2}$.

Presented results related to sample means, when Δ is in the vicinity of 1, are also in contrast with underlying physical mechanisms. Namely, as magnitudes of two dominant waves become approximately the same ($V_{1}\approx V_{2}$), i.e., when Δ≈1, it gets harder to discern the two rays [16]. However, Figure 1 shows that error in estimation of Δ (expressed by the difference between ${\hat{\Delta }}_{mean}$ and Δ and the dispersion of estimated values) in the vicinity of 1 is smaller than those obtained for moderate values of Δ. Accordingly, estimated values of Δ greater than 1, except for being useless for estimation of channel conditions, also lead to the false accurate sample means when Δ≈1, obtained by averaging potentially accurate values of ${\hat{\Delta }}_{j}$ smaller than 1 and unsubstantiated estimated values greater than 1.

On the other side, in the region of small and moderate values of Δ, the proposed estimator provides meaningful results. However, its usage leads to huge estimation error of parameter Δ, which is especially pronounced for small and moderate values of *K*.

Accordingly, due to the nonphysical definition of parameter Δ, although being analytically correct, $\hat{\Delta }$ ([4], Equation (16)) provides irrelevant results for certain combinations of parameters *K* and Δ, and huge errors in estimation of Δ for some others. Therefore, it is very desirable to derive estimators for physically justified Γ-based parameterization and to investigate their behavior in different channel conditions.

## 3. Moment-Based Estimators for the Γ-Parameterized TWDP Model

In order to derive moment-based estimators for the Γ-parameterized TWDP model, the expression for *n*-th moments of a signal envelope *r* given by ([4], Equation (6)) is expressed in terms of Γ, as:

(7) μn=E[rn]=n2!Ωn2(1+K)n22π∑m=0n2n2mKmm!∫02π1+2Γ1+Γ2cos(θ)mdθ

and for even values of *n*, obtained as a simple closed form expression:

(8) μn=n2!Ωn2(1+K)n2∑m=0n2n2mK1+Γ2m2F1−m,−m;1,Γ2m!,forn2∈N

where E(·) is the expectation operator.

Obviously, the *n*-th moment of signal’s envelope $\mu_{n}=E\left[r^{n}\right]$ depends on three unknown parameters: *K*, Γ, and Ω. Consequently, estimators of these parameters can be constructed from at least three different moments. Thereby, it is shown that estimation accuracy is the largest when the lower-order moments are used [4]. Accordingly, moment-based estimators of the Γ-parameterized TWDP model should be generated using second-, fourth-, and sixth-order moments, since only even moments can be obtained from (8). Thereat, in order to further reduce the estimation complexity, the impact of parameter Ω on *K* and Γ could be canceled out by properly defining ratios between the fourth- and the second-order as well as the sixth- and the second-order moments of an envelope [4]. It finally leads to the system of two equations:

(9) $$\frac{\mu_{4}}{\mu_{2}^{2}}=\frac{2+4K+K^{2}}{{\left(1+K\right)}^{2}}+{\left(\frac{2\Gamma }{1+\Gamma^{2}}\right)}^{2}\frac{K^{2}}{2{\left(1+K\right)}^{2}}$$

(10) $$\begin{array}{c} \frac{\mu_{6}}{\mu_{2}^{3}}=\frac{6+18K+9K^{2}+K^{3}}{{\left(1+K\right)}^{3}}+{\left(\frac{2\Gamma }{1+\Gamma^{2}}\right)}^{2}\frac{9K^{2}+3K^{3}}{2{\left(1+K\right)}^{3}} \end{array}$$

which could be solved for $\hat{K}$ and $\hat{\Gamma }$ if sample moments ${\hat{\mu }}_{n}=\frac{1}{N}\sum_{i=1}^{N}r_{i}^{n}$ are used instead of the ensemble averages $\mu_{n}$. In this regard, after (9) is inserted in (10), (10) can be expressed as: is expressed as:

(11) $$\begin{array}{c} \frac{{\hat{\mu }}_{6}}{{\hat{\mu }}_{2}^{3}}=3\frac{{\hat{\mu }}_{4}}{{\hat{\mu }}_{2}^{2}}-\frac{6{\hat{K}}^{2}+2{\hat{K}}^{3}}{{\left(1+\hat{K}\right)}^{3}}+{\left(\frac{2\hat{\Gamma }}{1+{\hat{\Gamma }}^{2}}\right)}^{2}\frac{3{\hat{K}}^{2}}{{\left(1+\hat{K}\right)}^{3}} \end{array}$$

i.e., as:

(12) $$\begin{array}{c} {\left(\frac{2\hat{\Gamma }}{1+{\hat{\Gamma }}^{2}}\right)}^{2}=\frac{6+2\hat{K}}{3}+\frac{{\left(1+\hat{K}\right)}^{3}}{3{\hat{K}}^{2}}\left(\frac{{\hat{\mu }}_{6}}{{\hat{\mu }}_{2}^{3}}-3\frac{{\hat{\mu }}_{4}}{{\hat{\mu }}_{2}^{2}}\right) \end{array}$$

Then, (12) is inserted in (9), transforming it into the following polynomial:

(13) $$\begin{array}{c} a{\left(1+\hat{K}\right)}^{3}+b{\left(1+\hat{K}\right)}^{2}+6\left(1+\hat{K}\right)-2=0 \end{array}$$

where *a* and *b* are defined as:

(14) $$\begin{array}{c} a=\frac{{\hat{\mu }}_{6}}{{\hat{\mu }}_{2}^{3}}-3\frac{{\hat{\mu }}_{4}}{{\hat{\mu }}_{2}^{2}}+2 \end{array}$$

(15) $$\begin{array}{c} b=6\left(1-\frac{{\hat{\mu }}_{4}}{{\hat{\mu }}_{2}^{2}}\right) \end{array}$$

By analyzing the discriminant of the polynomial (13), it can be shown that, for any values of *K* and Γ, the considered polynomial has one real root and one pair of complex conjugate ones. Thus, following the [17], one can find the real root of polynomial (13) as:

(16) 1+K^=−b+2Re(Z)3a

where Re(·) gives the real part of the complex number, and *Z* is defined as:

(17) $$\begin{array}{cc} Z & ={\left(p+\sqrt{q^{3}+p^{2}}\right)}^{1/3} \end{array}$$

where

(18) $$\begin{array}{cc} p= & 27a^{2}+27ab-b^{3} \end{array}$$

(19) $$\begin{array}{c} q=18a-b^{2} \end{array}$$

Considering the aforementioned, moment-based estimators of parameters *K* and Γ can be obtained as simple, closed form expressions:

(20) K^=−b+2Re(Z)3a−1

(21) $$\hat{\Gamma }=\frac{1-\sqrt{1-\left(\frac{6+2\hat{K}}{3}+\frac{{\left(1+\hat{K}\right)}^{3}}{3{\hat{K}}^{2}}\left(a-2\right)\right)}}{\sqrt{\frac{6+2\hat{K}}{3}+\frac{{\left(1+\hat{K}\right)}^{3}}{3{\hat{K}}^{2}}\left(a-2\right)}}$$

where parameters *a*, *b*, *Z*, *p*, and *q* can be easily calculated by inserting the second-, the fourth-, and the sixth-order sample moment of an envelope into the Equations (14), (15), (17), (18) and (19).

### Simulation-Based Performance Analysis of the Γ Estimator

**Figure 2:**
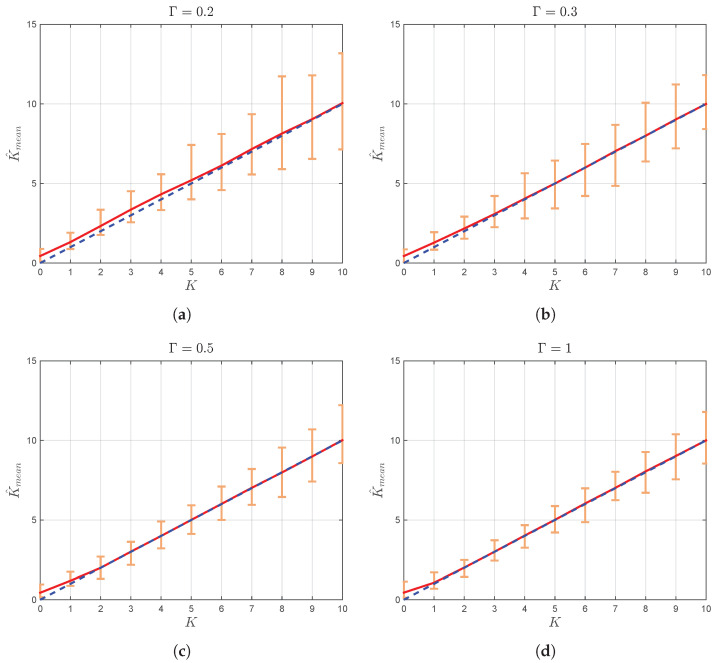
${\hat{K}}_{mean}$ (with absolute error bars) vs. *K* for (**a**) Γ=0.2, (**b**) Γ=0.3, (**c**) Γ=0.5, and (**d**) Γ=1. The solid line shows a linear regression fit to the data. Unit slope dashed line is illustrated as a benchmark.

**Figure 3:**
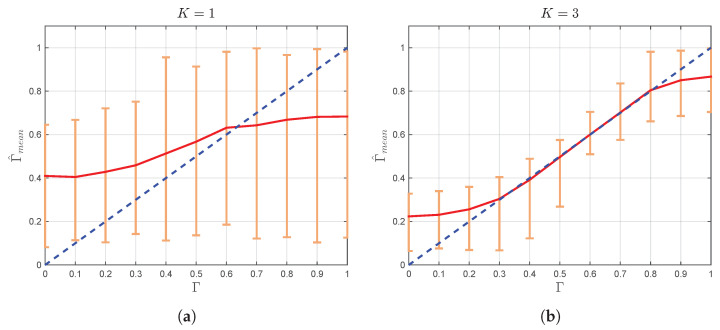

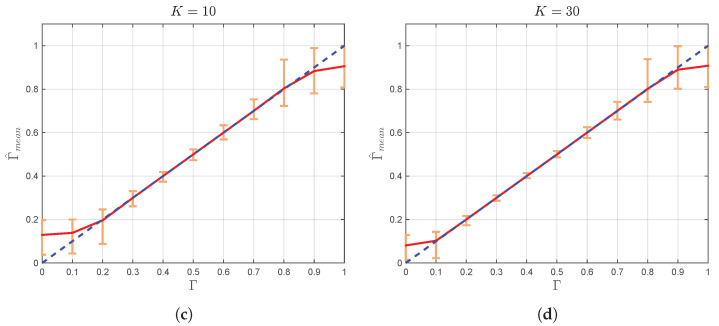
${\hat{\Gamma }}_{mean}$ (with absolute error bars) vs. Γ for (**a**) K=1, (**b**) K=3, (**c**) K=10, and (**d**) K=30. The solid line shows a linear regression fit to the data. The unit slope dashed line is illustrated as a benchmark.

After the expressions for $\hat{K}$ and $\hat{\Gamma }$ are derived, it is now necessary to investigate their performance in different channel conditions. For that purpose, Monte Carlo simulation is performed, and the obtained results are illustrated in Figure 2 and Figure 3.

Thereby, for each combination of *K* and Γ, where K={1,3,10,30} and Γ={0,0.1,0.2,…,1}, 500 realizations of the TWDP process with $N=10^{4}$ i.i.d. samples are generated and used in order to determine estimates ${\hat{K}}_{j}$ (20) and ${\hat{\Gamma }}_{j}$ (21), for j∈[1,500]. These values are used to calculate sample means ${\hat{K}}_{mean}$ and ${\hat{\Gamma }}_{mean}$ as ${\hat{K}}_{mean}=\left(1/500\right)\sum_{j=1}^{500}{\hat{K}}_{j}$ and ${\hat{\Gamma }}_{mean}=\left(1/500\right)\sum_{j=1}^{500}{\hat{\Gamma }}_{j}$. Figure 2 shows the boundaries where the estimates of parameter *K* for each realization of TWDP process are located with respect to the mean estimated value ${\hat{K}}_{mean}$, while Figure 3 illustrates the boundaries where each estimate of parameter Γ is located with respect to its mean estimated value.

From Figure 2, it can be observed that the estimator of parameter *K* given by (20) provides accurate results, especially in the region of medium and large values of *K* and Γ (e.g., K≥3 and Γ≥0.3 for $N=10^{4}$ samples), where ${\hat{K}}_{mean}$ is very close to *K*.

From Figure 3, it can be observed that Γ estimates, ${\hat{\Gamma }}_{j}$, are always smaller than one, which is in accordance with the physical mechanisms described in [16]. Consequently, as Γ approaches one, ${\hat{\Gamma }}_{mean}$ starts to increasingly deviate from Γ, causing an increase in error of its estimation. Accordingly, Figure 3 indicates that no anomalies ascertained for $\hat{\Delta }$ can be observed within the $\hat{\Gamma }$. It also can be observed that, for small values of *K* (in the vicinity of one), $\hat{\Gamma }$ provides accurate estimation only in the narrow range of Γ values close to 0.6. However, from the practical point of view, the results for relatively small values of *K* are irrelevant since, in that region, TWDP and Rayleigh distributions are almost identical. On the other side, the increment of *K* increases the range of Γ values where the estimation is accurate (more precisely, for a considered simulation with $N=10^{4}$ samples, ${\hat{\Gamma }}_{mean}$ is remarkably close to Γ for 0.2≤Γ≤0.8 and K≥3). From Figure 3, it can also be observed that, in the considered range, dispersion of estimated values (expressed by absolute error bars) is quite insignificant, indicating that derived estimator provides accurate estimates of Γ even for a relatively small number of samples (i.e., $10^{4}$).

## 4. AsV and CRB

To further assess the performance of proposed estimators, corresponding asymptotic variances for *K* and Γ, $AsV_{K}$ and $AsV_{\Gamma }$, are calculated from (20) and (21), as [4]:

(22) $$\begin{array}{c} AsV_{K}=g_{K}\mathrm{CvM} \end{array}$$

(23) $$\begin{array}{c} AsV_{\Gamma }=g{}_{\Gamma }\mathrm{CvM} \end{array}$$

where:

(24) $$\begin{array}{c} g_{K}={\left[\frac{\partial \hat{K}}{\partial {\hat{\mu }}_{2}},\frac{\partial \hat{K}}{\partial {\hat{\mu }}_{4}},\frac{\partial \hat{K}}{\partial {\hat{\mu }}_{6}}\right]}_{{\hat{\mu }}_{2}=\mu_{2},{\hat{\mu }}_{4}=\mu_{4},{\hat{\mu }}_{6}=\mu_{6}} \end{array}$$

(25) $$\begin{array}{c} g_{\Gamma }={\left[\frac{\partial \hat{\Gamma }}{\partial {\hat{\mu }}_{2}},\frac{\partial \hat{\Gamma }}{\partial {\hat{\mu }}_{4}},\frac{\partial \hat{\Gamma }}{\partial {\hat{\mu }}_{6}}\right]}_{{\hat{\mu }}_{2}=\mu_{2},{\hat{\mu }}_{4}=\mu_{4},{\hat{\mu }}_{6}=\mu_{6}} \end{array}$$

and CvM is a covariance matrix with elements:

(26) $$\begin{array}{c} {\left[\mathrm{CvM}\right]}_{ij}=\frac{1}{N}\left(\mu_{2i+2j}-\mu_{2i}\mu_{2j}\right),\;\mathrm{for}\;i,j=1,2,3 \end{array}$$

In addition, Cramer–Rao lower bounds are numerically calculated as [4]:

(27) $$\begin{array}{c} CRB_{K}={\left[I{\left(\theta \right)}^{-1}\right]}_{11} \end{array}$$

(28) $$\begin{array}{c} CRB_{\Gamma }={\left[I{\left(\theta \right)}^{-1}\right]}_{22} \end{array}$$

where the elements of Fisher Information Matrix I(θ), ${\left[I\left(\theta \right)\right]}_{ij}$, are determined as:

(29) $${\left[I\left(\theta \right)\right]}_{ij}=NE\left[\frac{\partial \ln f(r)}{\partial \theta_{i}}\frac{\partial \ln f(r)}{\partial \theta_{j}}\right],\;\mathrm{for}\;i,j=1,2,3$$

and where *N* is the number of observations, f(r) is the closed-form TWDP envelope PDF given by [14] [Equation (7)], and θ=[K,Γ,Ω]

**Figure 4:**
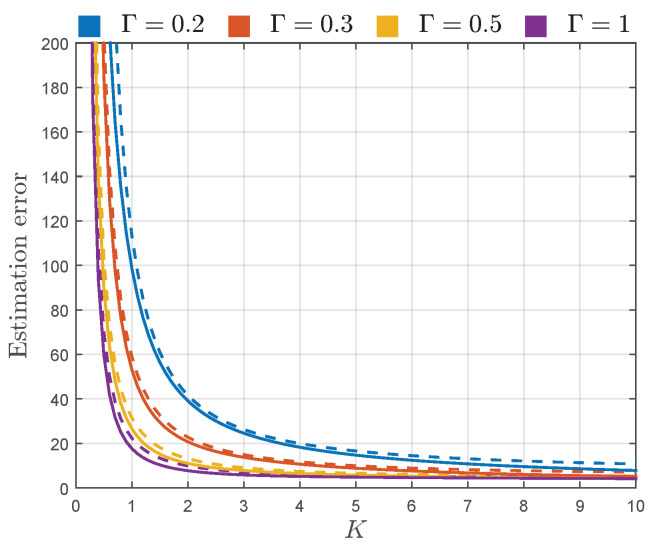
$\sqrt{CRB_{K}N/K^{2}}$ (solid line) and $\sqrt{AsV_{K}N/K^{2}}$ (dashed line) of $\hat{K}$ given by (20), for different values of Γ.

**Figure 5:**
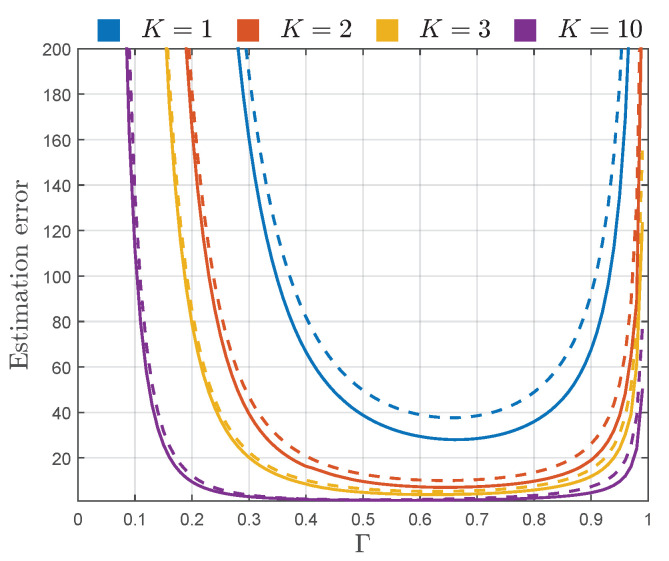
$\sqrt{CRB_{\Gamma }N/\Gamma^{2}}$ (solid line) and $\sqrt{AsV_{\Gamma }N/\Gamma^{2}}$ (dashed line) of $\hat{\Gamma }$ given by (21), for different values of *K*.

Thereby, following the approach presented in [4,7], instead of using CRB and AsV, the square root of CRB and AsV normalized to *N* and the true value of estimating parameter are used in estimator performance assessment. Thus, to assess the estimation error, the sqrt-normalized CRB and AsV of $\hat{K}$, $\sqrt{CRB_{K}N/K^{2}}$ and $\sqrt{AsV_{K}N/K^{2}}$, are plotted in Figure 4, and sqrt-normalized CRB and AsV of $\hat{\Gamma }$, $\sqrt{CRB_{\Gamma }N/\Gamma^{2}}$ and $\sqrt{AsV_{\Gamma }N/\Gamma^{2}}$, are plotted in Figure 5.

From Figure 4, it can be observed that the estimation error of *K* increases with the decrease of parameter *K*. Thereby, when the power of specular components $V_{1}^{2}+V_{2}^{2}$ is small with respect to the power of diffuse components $2\sigma^{2}$ (i.e., when the TWDP channel becomes Rayleigh-like), the error in estimation of *K* is very large. However, as the value of parameter *K* increases, i.e., as $V_{1}^{2}+V_{2}^{2}$ overrides $2\sigma^{2}$, the estimation of parameter *K* becomes very accurate. Figure 4 also shows that the error in estimation of *K* grows with the reduction of Γ, indicating that it becomes harder to accurately estimate *K* as the specular component with the magnitude $V_{2}$ becomes less significant with respect to one with the magnitude $V_{1}$.

From Figure 4, it can also be observed that the values of sqrt-normalized $AsV_{K}$ are remarkably close to the sqrt-normalized $CRB_{K}$ for the entire considered range of parameters *K* and Γ, indicating almost asymptotic efficiency of the proposed estimator of a parameter *K*.

Figure 5 shows that the estimation error of parameter Γ behaves similarly as the estimation error of $\hat{K}$, with respect to *K* and Γ. Hence, the estimation of Γ deteriorates with the reduction of *K*, i.e., as the power of diffuse components becomes more significant with respect to $V_{1}^{2}+V_{2}^{2}$. The estimation error of $\hat{\Gamma }$ is large for small values of Γ, indicating that it is hard to estimate the values of Γ when $V_{2}$ is insignificant with respect to $V_{1}$. For moderate values of Γ, $\hat{\Gamma }$ given by (21) starts to provide pretty accurate results, especially for large values of *K*. However, as Γ approaches one, estimation of Γ becomes more inaccurate. In these conditions, the magnitudes of specular components, $V_{1}$ and $V_{2}$, become similar. Considering the aforementioned and the fact that the phase difference between these components is uniform, the probability of destructive superposition of specular components becomes more likely, making their overall magnitude often insignificant. Thus, as Γ approaches one, it gets harder to accurately determine the value of $V_{2}/V_{1}$, especially when the power of diffuse components is large with respect to $V_{1}^{2}+V_{2}^{2}$.

When it comes to the observation of the proximity of the proposed Γ estimator to its CRB, it can be concluded from Figure 5 that the values of the sqrt-normalized $AsV_{\Gamma }$ are remarkably close to the sqrt-normalized $CRB_{\Gamma }$ for K≥2 in the entire range of Γ, making the proposed estimator asymptotically efficient for the considered values of *K*.

Accordingly, except for providing estimation errors significantly close to the corresponding CRBs, moment-based estimators (20) and (21) provide accurate estimates obtained from a relatively small number of samples, which can be clearly observed from Figure 4 and Figure 5. For example, if we assume that the sufficient accuracy of estimation process is 20%, it can be achieved with $N=10^{4}$ samples for Γ∈[0.3,0.9] and K≥3 (obtained by multiplying estimation errors $\sqrt{AsV_{K}N/K^{2}}$ and $\sqrt{AsV_{\Gamma }N/\Gamma^{2}}$ by $1/\sqrt{N}$).

If necessary, the estimation accuracy in the determined region can be increased, or the region itself can be further expanded, by involving more samples within the estimation process (e.g., by employing $N=10^{6}$ samples, relative estimation error in the considered region of parameters *K* and Γ could be reduced to 2%, or the estimation error of 20% could be achieved for the wider range of *K* and Γ, i.e., K≥3 and Γ∈[0.16,0.99]). In this way, the procedures used to create Figure 4 and Figure 5 can be used to determine the number of samples needed to obtain desired estimation accuracy within the desired range of parameters *K* and Γ.

## 5. Comparison of Moment-Based Estimators for the Δ- and Γ-Parameterized TWDP Model

**Figure 6:**
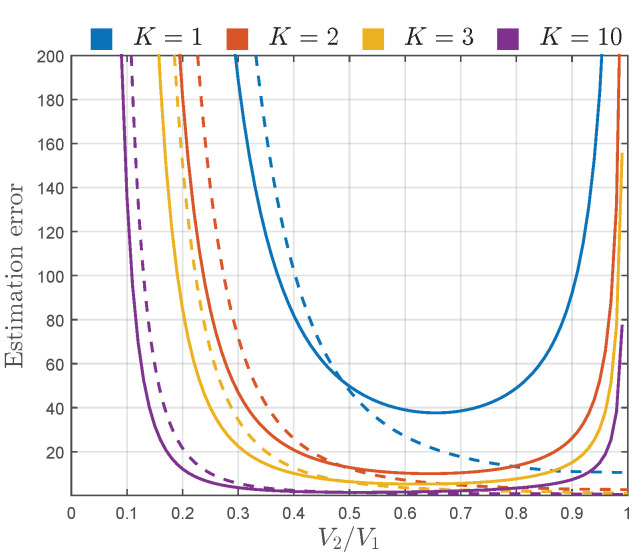
$\sqrt{AsV_{\Gamma }N/{\left(V_{2}/V_{1}\right)}^{2}}$ of $\hat{\Gamma }$ (solid line) and $\sqrt{AsV_{\Delta }N/{\left(V_{2}/V_{1}\right)}^{2}}$ of $\hat{\Delta }$ (dashed line), for different values of *K*.

In order to observe qualitative differences between Γ- and Δ-based parameterization and to gain more precise insight into the relation between estimation errors for considered parameterizations, $AsV_{\Delta }$ and $AsV_{\Gamma }$ are normalized to the same parameter ratio $V_{2}/V_{1}$ and presented in Figure 6. This enables us to compare absolute values of AsV for Δ and Γ and to discover differences in their estimation errors for considered ratios between $V_{1}$ and $V_{2}$. Figure 6 shows that, for $0\leq V_{2}/V_{1}\leq 0.5$ (which corresponds to 0≤Δ≤0.8), estimation error of parameter Γ is up to two times smaller than the error obtained for Δ parameter estimation. On the other side, for $0.5<V_{2}/V_{1}<0.8$ (i.e., 0.8<Δ<0.96) and K≥3, there is no significant difference in accuracy of $\hat{\Delta }$ and $\hat{\Gamma }$. Finally, for $V_{2}/V_{1}\in \left[0.8,1\right]$, error in estimation of Γ starts to increase with the increment of $V_{2}/V_{1}$, thus being in line with the model’s physical mechanisms. On the contrary, in the considered region, Δ-based parameterization provides false accurate results, obtained by also considering values of $\hat{\Delta }$ greater than one in calculation of $AsV_{\Delta }$.

Accordingly, in the region of Δ≤0.8, i.e., Γ≤0.5, estimation accuracy is significantly improved by using Γ-based parameterization instead of Δ-based, while in the region of Δ≈1, i.e., Γ≈1, Γ-based parameterization prevents the occurrence of nonphysical solutions obtained by estimating parameter Δ.

Except for benefits of Γ-based parameterization observed with respect to $\hat{\Gamma }$, it also enables reduction of the estimation error of a parameter *K*, for a much wider set of values of a parameter, which reflects the relation between $V_{1}$ and $V_{2}$. Namely, based on the expression of parameter *K* given in terms of Δ and the results presented in [14] [Figure 2], it can be concluded that $K/K_{Rice}$ is almost constant for the entire range of small and medium values of Δ, implying that values of $V_{2}\in \left[0,V_{1}/2\right]$ make almost no impact on the value of parameter *K*. This causes quite pronounced errors in estimation of *K* for the entire range of small and medium values of Δ (i.e., 0≤Δ<0.5), which can be clearly observed from [4] [Figure 1]. On the contrary, Figure 4 shows that, when *K* is expressed in terms of Γ, no such anomaly can be observed. In these circumstances, errors in estimation of *K* are huge only for small values of Γ (i.e., Γ<0.2).

## 6. Conclusions

In this paper, the problem of TWDP parameters’ estimation has been investigated in depth. The investigation revealed that the existing moment-based estimators of conventional TWDP parameters are not able to provide accurate estimations for various combinations of their values, due to a nonphysical definition of parameter Δ. Accordingly, in this paper, moment-based estimators for physically justified parameters *K* and Γ are derived. It is shown that derived estimators provide estimates from $10^{4}$ samples with the estimation error smaller than 20%, when parameters *K* and Γ are in the range K≥3 and 0.3≤Γ≤0.9. This indicates that the parameters *K* and Γ can be efficiently estimated using derived expressions within the range of these parameters expected to be obtained in the mmWave band, even from a relatively small number of samples. Since Γ-based estimators enable us to gain precise insight into the ratios between two specular and specular to diffuse components in the wide varieties of propagation conditions, simultaneously reducing estimation errors with respect to Δ-based parameterization, it is recommended to adopt parameters *K* and Γ as the only relevant parameters for a description of TWDP fading and to revise the existing measurement-based results related to the estimation of TWDP parameters in specific propagation conditions.

## Data Availability

Not applicable.
